# Methadone-based Multimodal Analgesia Provides the Best-in-class Acute Surgical Pain Control and Functional Outcomes With Lower Opioid Use Following Major Posterior Fusion Surgery in Adolescents With Idiopathic Scoliosis

**DOI:** 10.1097/pq9.0000000000000336

**Published:** 2020-07-27

**Authors:** Jian Ye, Karen Myung, Senthil Packiasabapathy, Jeffrey S. Yu, Joseph E. Jacobson, Stephanie C. Whittaker, Peter Castelluccio, Meghan Drayton Jackson, Senthilkumar Sadhasivam

**Affiliations:** From the * Department of Anesthesia, Riley Hospital for Children at IU Health, Indiana University School of Medicine, Indianapolis, IN; † Department of Orthopedic Surgery, Riley Hospital for Children at IU Health, Indianapolis, IN; ‡ Indiana University School of Medicine, Indianapolis, IN; § Department of Biostatistics, Indiana University School of Medicine, Indianapolis, IN; ¶ Department of Pediatrics, Indiana University School of Medicine, Ind.

## Abstract

**Introduction:**

Posterior spinal fusion for idiopathic scoliosis is extremely painful, with no superior single analgesic modality. We introduced a methadone-based multimodal analgesia protocol, aiming to decrease the length of hospital stay (LOS), improve pain control, and decrease the need for additional opioids.

**Methods:**

We analyzed 122 idiopathic scoliosis patients with posterior instrumented spinal fusion. They were matched by age, sex, surgeon, and the number of levels fused before and after the implementation of the new protocol. This analysis included 61 controls (intrathecal morphine, gabapentin, intravenous opioids, and adjuncts) and 61 patients on the new protocol (scheduled methadone, methocarbamol, ketorolac/ibuprofen, acetaminophen, and oxycodone with intravenous opioids as needed). The primary outcome was LOS. Secondary outcomes included pain scores, total opioid use (morphine milligram equivalents), time to a first bowel movement, and postdischarge phone calls.

**Results:**

New protocol patients were discharged earlier (median LOS, 2 days) compared with control patients (3 days; *P* < 0.001). Total inpatient morphine consumption was lower in the protocol group (*P* < 0.001). Pain scores were higher in the protocol group on the day of surgery, similar on postoperative day (POD) 1, and lower by POD 2 (*P* = 0.01). The new protocol also reduced the median time to first bowel movement (*P* < 0.001), and the number of postdischarge pain-related phone calls (*P* < 0.006).

**Conclusion:**

Methadone-based multimodal analgesia resulted in significantly lower LOS compared with the conventional regimen. It also provided improved pain control, reduced total opioid consumption, and early bowel movement compared with the control group.

## INTRODUCTION

Posterior spinal fusion (PSF) for adolescent idiopathic scoliosis is often excruciatingly painful procedure.^1^ Many adolescents have heightened pain perception due to their immature emotional state.^2^ Inadequate management of acute postoperative pain can delay functional recovery and prolong the length of hospital stay (LOS). It can also lead to chronic persistent postsurgical pain (36% prevalence) and prolonged opioid use (10% prevalence) after PSF.^3,4^

Many postoperative analgesic strategies are available for patients undergoing PSF, but no single modality has been superior. Patient-controlled analgesia (PCA) with intravenous (IV) opioids has been standard in many centers, but opioids have undesirable side effects, including respiratory depression. Epidural or intrathecal administration of local anesthetics have opioid-sparing analgesia.^5–8^ Unfortunately, epidural analgesia has a high failure rate and can interfere with postoperative neurologic evaluation. Intrathecal morphine is associated with urinary retention, itching, and, rarely, respiratory depression.

There is a growing body of literature to support the intraoperative use of methadone—a long-acting opioid.^9^ Methadone is efficacious for complex spine surgery in adults.^10,11^ Some studies suggest postoperative respiratory monitoring is prudent due to high incidence (36.8%) of respiratory depression when used with PCA hydromorphone.^12^ However, there is limited evidence for the effectiveness and safety of perioperative methadone in children. Methadone has been effective for postoperative pain in younger children and those undergoing Nuss procedure for surgical correction of pectus excavatum.^13,14^ There is only limited evidence of methadone for PSF is available in children. In a randomized control study of 60 adolescents undergoing PSF, intraoperative methadone decreased intraoperative opioid requirements.^15^ In a small study of 31 adolescents that received a single relatively larger dose of intraoperative methadone (0.1–0.3 mg/kg; maximum 20 mg), there was no reduction in postoperative opioid consumption.^16^ This result could be related to the relatively shorter half-life of methadone in children compared with adults.^17^ Moreover, a single larger dose of intraoperative methadone in adolescents had a high incidence of respiratory depression after PSF.^12^ A single dose of intraoperative methadone, as part of a multimodal analgesia regimen, improved pain scores with modest shortening in LOS without significant reduction in opioid consumption on POD 1 and 2.^18^

Multimodal analgesia with opioids, nonopioid analgesics, and muscle relaxants reduce opioid usage, minimize opioid-related side effects, facilitate the transition from IV to oral administration, and permit earlier hospital discharge.^18–21^ Muscle spasm contributes to a significant amount of pain after PSF. Methocarbamol, in particular, has been proven to be safe and effective for acute lower back pain.^22–24^ Nonsteroidal anti-inflammatory drugs (NSAIDs) are safe and effective in pediatric orthopedic surgery.^25–27^ Acetaminophen is a centrally acting cyclo-oxygenase inhibitor and an effective opioid-sparing analgesic in children.^28,29^

We hypothesized that a multimodal analgesia regimen, including multiple and small doses of methadone and nonopioid analgesics and adjuncts, would promote earlier hospital discharge, improve pain scores, and lower opioid consumption in adolescents undergoing PSF.

## METHODS

### Context

We performed this quality improvement initiative at the Riley Hospital for Children, a tertiary care hospital. Before implementing the new protocol, all PSF patients received intrathecal morphine and postoperative morphine PCA with the median LOS of 3 to 4 days. We implemented the new Acute Pain Service (APS) protocol (Table 1) in September 2017 to enhance recovery and decreasing LOS. The decision was made a priori to collect outcome data, including LOS, pain scores, opioid consumption, and opioid-related adverse effects.

**Table 1.**
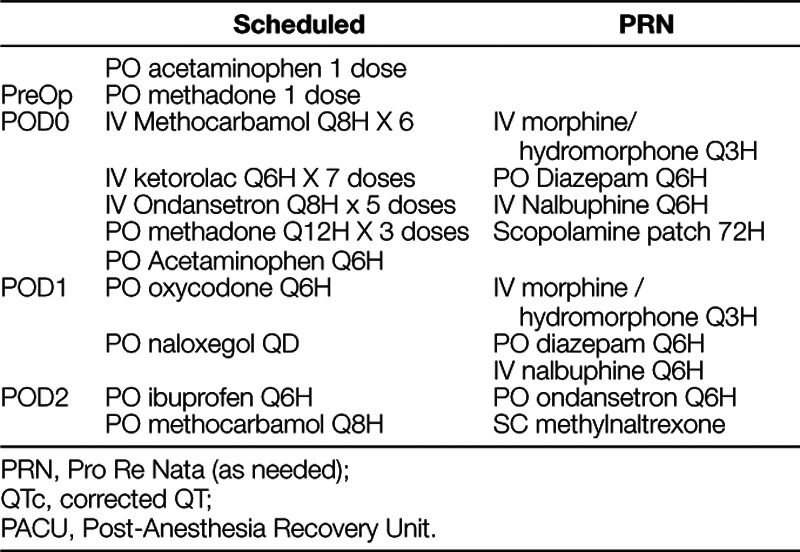
Acue Pain Service (APS) Intervention Protocol

### Design

We analyzed outcomes using a retrospective matched cohort design after approval by the institutional review board (with waived informed consent.) We compared the APS intervention cohort with the historical matched controls who received PSF before protocol implementation. All subjects were operated on by the same pediatric spine surgeon.

### Enrollment criteria

The baseline period (November 2012 to August 2017) included 102 patients who had PSF; we selected 61 matched patients. The intervention (APS protocol) period (September 2017 to January 2019) included 72 PSF patients; however, only 61 patients received methadone and the full interventions due to drug shortages. We matched patients on age, sex, and the number of vertebral levels fused (Table 2) since these factors contribute to overall opioid usage.^3^ A single pediatric spine surgeon performed all procedures. We excluded subjects with neuromuscular scoliosis, congenital QT prolongation, and allergy to any of the protocol drugs (ketorolac, methocarbamol, methadone, or acetaminophen) during the intervention period.

**Table 2.**
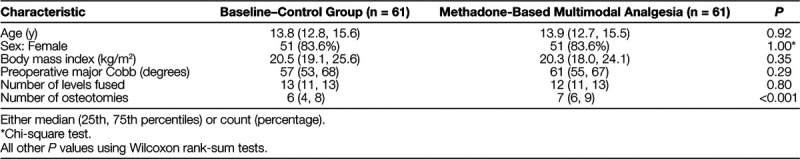
Demographics

### Standardized anesthesia management

Anesthesia management was consistent during the study. We standardized the intraoperative anesthesia technique for both groups. After induction and endotracheal intubation, we obtained venous access and arterial line. The patients were placed on total IV anesthesia with propofol (75–200 µg/kg/min), remifentanil (0.1–0.5 µg/kg/min), and ketamine (0.25–1 mg/kg/h) to facilitate intraoperative neuromonitoring.

### Baseline period analgesia management

Before the protocol was implemented, the patients were given intrathecal morphine by the surgeon at a dose of 5 µg/kg intraoperatively. Postoperatively, they were placed on oral gabapentin (100 mg per dose) 3 times a day. They received a morphine PCA on POD0 and transitioned to oxycodone or hydrocodone on POD1 with IV morphine as needed for breakthrough pain. Patients received ketorolac, acetaminophen, and diazepam as needed.

### Intervention period analgesia management (APS protocol)

The APS protocol (Table 1) was developed based on evidence,^9–11,15,16,18^ and the authors’ clinical expertise using multiple small doses of methadone and multimodal analgesia in adolescents undergoing major inpatient surgery. Preoperatively, patients received oral acetaminophen (15 mg/kg, maximum 650 mg). As this multimodal protocol with scheduled ondansetron was new at our institution, we obtained an electrocardiogram (ECG) preoperatively in the orthopedic clinic to screen for baseline QT prolongation.

Intraoperatively, the APS group received a small dose of IV methadone (0.1 mg/kg, maximum 5 mg). Single intraoperative doses (0.1–0.3 mg/kg) in adolescents have a higher incidence of adverse effects and lack of sustained analgesia.^16^ We used a small dose of methadone (0.1 mg/kg, maximum 5 mg doses) intraoperatively and repeated every 12 hours until discharge to minimize adverse effects and sustain analgesia.

Postoperatively, patients received 3 more doses of methadone (0.1 mg/kg, maximum of 5 mg) every 12 hours starting on the evening of the day of surgery (POD0). An ECG was obtained on POD1 to verify that there was no significant QT prolongation (> 460 ms). Methocarbamol was given every 8 hours with the first dose given in the post-anesthesia recovery unit. Ketorolac was scheduled for the first 3 days every 6 hours, with the first dose given at 8:00 PM on POD0 (or immediately after surgery if before 2:00 PM). On POD2, we stopped ketorolac and started ibuprofen before discharge. We started oral acetaminophen every 6 hours, starting on POD1. We used IV hydromorphone as needed for breakthrough pain. Patients did not receive PCA opioids. Patients received oral oxycodone on POD 1 around noon when tolerating oral diet. Patients received scheduled ondansetron every 8 hours for the first 48 hours after surgery as well as scheduled naloxegol, a peripherally acting μ-opioid receptor antagonist, to prevent opioid-induced constipation (Table 1).

Postoperatively, the APS team managed acute surgical pain by placing all analgesic orders and assessing patients daily. Before discharge, the APS team performed medication management education with the patient, parents, and nursing staff. Per Indiana state regulations, patients received the opioid prescription for 7 days postdischarge.

### Bowel Regimen

Postoperative constipation or ileus is a common problem in the scoliosis population, both due to the nature of the surgery and the frequent use of opioids. The bowel regimen was unchanged between the baseline and the intervention period, and it included polyethylene glycol (17 g) per day, starting 5 days preoperatively and continuing after surgery. On the morning of POD1, patients received a glycerin suppository and a dose of magnesium hydroxide. Patients who had no bowel movement by the evening of POD1 received an enema. During the intervention period, patients received additional oral Naloxegol.^30^

### Physical Therapy

During the baseline and intervention periods, all patients received similar postoperative standardized physical therapy (PT) and rehabilitation measures, including getting out of bed and room with assistance.

### Discharge Readiness

Hospital discharge criteria for both groups included: (1) able to take oral food, including oral analgesics without nausea/vomiting; (2) pain controlled by oral analgesics; (3) regular bowel movements; (4) ambulation with minimal help and without significant pain; and (5) no surgical complications (eg, bleeding, wound infection).

### Outcome Measures

The primary outcome was time to hospital discharge (days). Secondary outcomes included opioid consumption (in morphine milligram equivalents [MME]) on each postoperative day and in total, pain scores (taken on a 0–10 Numerical Rating Scale for pain and averaged over each postoperative day), opioid-related side effects (measured by time to first bowel movement, as constipation is a significant issue), and postdischarge pain-related phone calls.

### Statistical Analysis

**Fig. 1.**
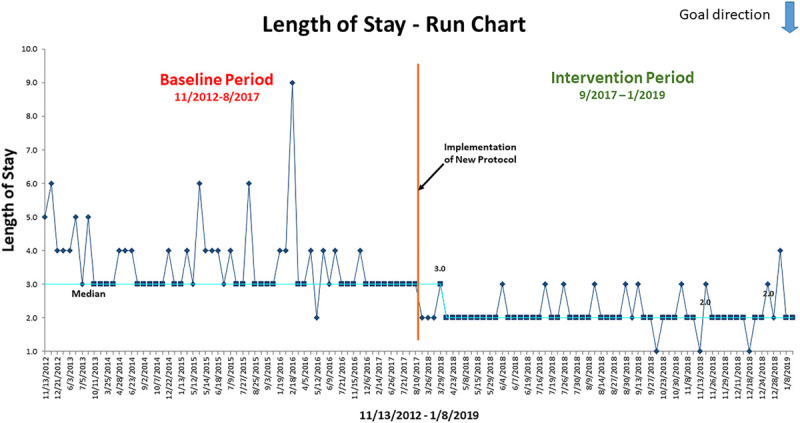
Length of hospital stay (LOS) time series run chart. The run chart illustrates patients treated with the APS protocol during the intervention period had shorter hospital stays than patients in the baseline period. The median length of stay was 2 days during the intervention period, a decrease from the baseline median of 3 days.

**Fig. 2.**
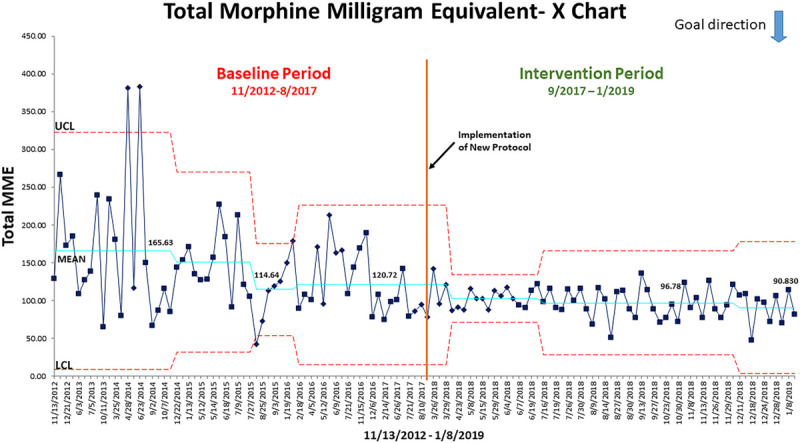
Postoperative opioid use—X chart. The mean postoperative opioid usage in terms of Morphine Milligram Equivalents ((MME) is plotted. The upper (UCL) and lower (LCL) control limits are the 3 standard deviations from the mean. The wider control limits in the baseline period highlight that the process had a lot of variation, including 2 patients that were outliers outside of the UCL. The total MME was trending down just before the new protocol implementation as we developed the new standardized protocol. The new protocol intervention added to that decline and, most significantly, the control limits narrowed, indicating less variability and more reliability in the process for similar outcomes.

We used QI Macros for Excel Version 2019.06 to statistically analyze the impact of our intervention on LOS (Fig. 1) and total MME (Fig. 2). The LOS is displayed in a run chart, and the total MME is displayed in an X-chart. We compared categorical variables using the Chi-square test. We used the Wilcoxon rank-sum test for comparison of medians. We used a mixed model for repeated measures for comparing means of normally distributed variables and adjusted for multiple testing via Sidak correction.

## RESULTS

We included a total of 122 patients—61 patients in the baseline period and 61 in the intervention period (Table 2). One notable change in the APS protocol, immediately after implementation, was the removal of pregabalin administration. We initially included pregabalin in the regimen due to its neuromodulating properties,^31^ and stopped its use after the first 3 patients due to significant urinary retention observed in these patients. Other minor changes included switching from IV methadone to oral (and back to IV), and switching to granisetron from ondansetron for a short while due to drug shortages.

### Primary Outcome

Patients treated with the APS protocol had significantly shorter hospital stays than patients in the baseline period (Table 3, Figure 1). The median length of stay was 2 days. Most patients discharged essentially on POD2 AM, with some on POD1 PM—a decrease from the baseline median of 3 days (Fig. 1) (*P* < 0.001).

**Table 3.**
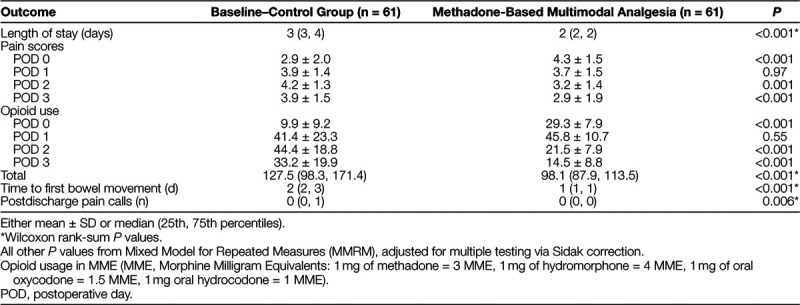
Results of Implementing Multimodal Analgesia Protocol in Idiopathic Scoliosis Patients Undergoing Spinal Fusion Surgery

### Secondary Outcomes

The mean opioid usage (MME) (29.3 vs 9.9 mg) for patients in the intervention period was higher on POD0 compared with the control group (*P* < 0.001, Table 3, likely due to earlier ambulation and the higher scheduled dosing of opioids on the APS protocol. Mean opioid consumption was similar on POD1 for the 2 groups, but 50% lower for the APS group than the control group on POD2 (21.5 vs 44.4 mg; *P* < 0.001). Total opioid consumption was approximately 30% lower for the APS group (mean of 98 mg in the APS group vs 128 mg in the control group; *P* < 0.001). In Figure 2, the wider control limits in the baseline period highlight that the process had a lot of variation, including 2 patients that were outliers. The total MME was already trending down before the new protocol implementation, and the intervention added to that decline. The control limits narrowed significantly, indicating less variability and more reliability in the process for similar outcomes.

The mean pain scores were higher in the APS group than controls on POD0 (4.3 vs 2.9; *P <* 0.001; Table 3), likely due to earlier ambulation in the APS group and/or the beneficial effects of intrathecal morphine in the control group. Pain scores were similar on POD1 and lower on POD2 for the APS group (mean 3.2 vs 4.2 for the control group; *P* = 0.001). The overall trend showed decreasing pain scores in the APS protocol group and increasing pain scores in the control group over the first 3 days after surgery before discharge.

The median time to first bowel movement was significantly shorter in the APS group compared with the control group (1 day vs 2 days; *P* < 0.001). The number of postdischarge pain-related phone calls was significantly less in the APS intervention period compared with the baseline period (*P* < 0.006; Table 3).

### Adverse Effects

No significant opioid-related adverse effects (especially respiratory depression) *were observed* in the APS group. The first postoperative bowel movement was achieved 1 day earlier during the intervention period (Table 3). The mean corrected QT interval increased from baseline to POD1 (416–431 ms), remaining within normal limits (< 440 ms).

## DISCUSSION

New methadone-based multimodal analgesic protocol significantly enhanced the recovery from PSF for patients with idiopathic scoliosis. Methadone-based multimodal analgesia significantly reduced hospital stays, postoperative pain scores, total opioid consumption, and opioid-associated side effects, including constipation. The median LOS in our study population (2 days) was significantly shorter than the national average for PSF and most of the published studies on multimodal analgesia protocols.^18,32^ This observation is particularly significant in reducing the cost of care in a value-based model and reducing potential risks for hospital-acquired infections following major spinal instrumentation. This shorter hospitalization translates to at least $10,000 health care cost savings per patient in 2020 U.S. dollars.

In our protocol, we chose lower intraoperative doses of methadone to avoid adverse effects reported with higher doses in adults and children. We also dosed methadone every 12 hours to compensate for rapid clearance in children, sustain the analgesic effects, and decrease the necessity for short-acting opioids. We were able to significantly lower overall opioid use, pain scores, and opioid-related adverse effects while achieving early ambulation and the shortest reported LOS following spine fusions. In all study patients receiving the lower dose of methadone, routine ECGs revealed no significant QT prolongation or arrhythmias requiring intervention. We will discontinue routine ECGs to reduce costs further if methadone does not prolong corrected QT significantly in a larger cohort.

During the implementation of the protocol, we also noted other subjective improvements, including earlier mobilization from bed to chair, more productive PT sessions, and a better overall sense of patient comfort and well-being without grogginess. Given the heavy pain burden these surgeries impose on adolescents even years later,^33^ there is certainly benefit in giving them a more pleasant perioperative experience.

It is important to note that this was also a multidisciplinary effort between the orthopedic and the anesthesia acute pain service. A dedicated pediatric acute pain service is highly valuable,^34^ but it is frequently difficult to implement—even in the setting of large pediatric hospitals.^35^ The constant presence of the pain service has reassured patients and their families, improved inpatient acute pain management, and pain management after discharge due to the education on the discharge medication calendar.

Our experience with the methadone-based standardized multimodal analgesia protocol for idiopathic scoliosis surgery led us to implement similar protocols in children undergoing other painful procedures such as pectus excavatum repair, with similar benefits. Our patients have fared so well on the methadone-based multimodal analgesia protocol that we have discussed a goal of routine POD1 discharge following major spine fusions in adolescents with idiopathic scoliosis. Several patients in the methadone-based protocol group, about 25%, were discharged home the day after spinal fusion surgery (POD1). Achieving POD1 discharge for the average patient would drastically change the landscape of adolescent scoliosis surgery, leading to substantial cost savings, about $10,000 per patient.

Methadone itself is less expensive compared with the opioid PCA. Acquisition cost of PCA hydromorphone syringe (75 mcg/mL × 30 mL syringe) is $18, while methadone 5 mg is $1.58 (>10-fold cheaper). Typical pharmacy charges are about 10-fold higher than the acquisition costs. Some institutions add a charge for the use of the PCA pump ($200–500 per patient). Our research will enable institutions to provide cost-effective and safe pediatric-enhanced surgical recovery protocols for value-based care under bundled payments.

The new protocol also reduced opioid use and lowered postoperative pain scores. We are following PSF patients for chronic persistent pain and long-term opioid use. Effective management of acute postoperative pain and use of ketamine and methadone perioperatively had decreased incidence of persistent surgical pain to about 5% in the last 2 years. This finding is substantially less than the 36% incidence reported in adolescents receiving nonmethadone-based multimodal acute postoperative pain management.^36^ This result is significant as we face a national opioid epidemic.

There are a few limitations to our study. It was a retrospective chart review with limited data on subjective measures such as patient satisfaction and physical mobility. However, the feedback from physical therapists and inpatient nursing staff has been consistently positive in terms of patients’ comfort, cooperation, and early ambulation without drowsiness (frequently observed with the previous pain regimen). Another limitation is that the APS analgesic protocol had minor changes during implementation due to drug shortages (eg, IV ondansetron in place of IV granisetron, oral methadone in place of IV methadone for a brief duration). The oral bioavailability of methadone is 86%, and it is considered interchangeable with IV methadone. One of the more noteworthy modifications was removing pregabalin from the protocol—almost immediately after its implementation. Due to the evidence supporting the use of neuromodulators in the setting of postoperative pain for spine surgery,^31^ pregabalin had been part of the protocol initially. However, we experienced significant postoperative urinary retention in 3 patients with pregabalin, which resolved once we stopped it.

Although we matched for known variables (age, sex, surgeon, and the number of laminectomies), there is potential for sampling bias secondary to our effort to match cases individually. Due to the study design, temporal changes may have occurred over time. We made efforts to generalize all other aspects of patient management (standardization of anesthesia, surgical management, and PT). Before the protocol implementation, we examined discharge data to screen for random fluctuation and eventual regression to mean after implementation. Large prospective multicenter trials are needed to validate our findings externally.

## CONCLUSIONS

In conclusion, a new multimodal analgesic protocol following posterior spinal fusion surgery led to significantly reduced LOS, improved inpatient postoperative pain control, lower total opioid consumption, and opioid-related adverse effects following posterior spine fusion in adolescents with idiopathic scoliosis. Implementing this methadone-based multimodal analgesia protocol in other institutions may allow for enhanced recovery in children following this extremely painful surgery while lowering hospitalization costs and reducing opioid use. The protocol may lower the potential for opioid-associated adverse effects, including persistent opioid use and possibly reduce the incidence of persistent surgical pain. To replicate and externally validate this protocol in other centers, we need a large multicenter study in PSF for more complicated neuromuscular spines. We are currently evaluating the protocol for adolescents undergoing Nuss bar repairs for pectus excavatum, in addition to a continuous thoracic epidural analgesia.

## DISCLOSURE

The authors have no financial interest to declare in relation to the content of this article.

## PRESENTATIONS

We presented this work at the 2019 annual Pediatric Orthopaedic Society of North America (POSNA) and the 2020 annual meeting of the Society for Pediatric Anesthesia (SPA).

## ACKNOWLEDGMENTS

The authors acknowledge Janelle S. Renschler, DVM, PhD (Department of Anesthesia, Indiana University, Indianapolis, IN), for assistance with medical writing and editing, which was funded by Indiana University following Good Publication Practice (GPP3) guidelines (http://www.ismpp.org/gpp3). The Department of Anesthesia resources funded this study at Indiana University School of Medicine, Indianapolis. The Eunice Kennedy Shriver National Institute of Child Health & Human Development of the National Institutes of Health under Award Numbers, R01HD089458 (PI: S.S.), R21HD094311(PI: S.S.), and R01HD096800 (PI: S.S.) supported Dr. Sadhasivam’s effort. The content is solely the authors’ responsibility and does not necessarily represent the official views of the National Institutes of Health. The departments of Anesthesia and Clinical Pharmacology at Indiana University School of Medicine (IUSM) provided salary support for the authors.
